# Histological Changes and Immunohistological Analysis of Interstitial Cells of Cajal in Pelvi-Ureteric Junction Obstruction: Correlation With Preoperative Renal Function

**DOI:** 10.7759/cureus.87970

**Published:** 2025-07-15

**Authors:** Priya S Patil, Santosh V Patil, Manisha R Dhobale, Nitin R Mudiraj

**Affiliations:** 1 Anatomy, Bharati Vidyapeeth (Deemed to Be University) Medical College and Hospital, Sangli, IND; 2 Pediatric Surgery, Ushahkal Abhinav Institute of Medical Sciences, Sangli, IND

**Keywords:** fibrosis, immunohistochemistry, interstitial cells of cajal, masson trichrome stain, pelvi-ureteric junction obstruction, renal scan

## Abstract

Introduction: A decrease in smooth muscle cells at the pelvi-ureteric junction (PUJ), along with abnormal muscle orientation, collagen deposition, and a reduction in Cajal cells, is proposed as the primary cause of obstruction at the PUJ. The findings on histology and the density of interstitial cells of Cajal (ICCs) in patients operated for pelvi-ureteric junction obstruction (PUJO) show variable observations. Clinicians often face a dilemma during the diagnosis and management of patients with PUJO. Hence, to address this knowledge gap, a study of the detailed microanatomy of PUJO and the number of ICCs has been undertaken. A correlation of these parameters with preoperative renal function will help clinicians during postoperative follow-up and prediction of the surgical outcome.

Methodology: The study was carried out on resected specimens from all 54 patients who underwent surgery for PUJO during the study period. The gross features were noted, and bits were taken from the obstructed middle part of the PUJ and the normal distal surgical margin (DSM). The sections were stained with routine hematoxylin and eosin (H&E), Masson's trichrome for collagen, and immunohistochemistry using the c-Kit antigen for ICCs.

Results: The study showed a male preponderance of PUJO and antenatal detection in 36 (66.7%) cases. Histological evaluation on H&E staining revealed a narrowed ureteric lumen, a lining epithelium that was either thickened or normal, hypertrophied smooth muscle, and increased fibrosis, most prominently at the PUJ. In some cases, the wall showed increased vascularity, lymphocytic infiltration, prominent nerve bundles, muscle hypertrophy, and fibrosis. These findings were more marked at the obstructed segment. In a few cases, the wall showed atrophied smooth muscles and severe perifascicular fibrosis, which was more prominent at the PUJ. There was a significant difference in the thickness of the lamina propria and muscle layer at PUJ and DSM in the older age group. Masson's trichrome stain is useful for detecting and grading fibrosis at the PUJ due to its differential staining properties. It showed higher grades of fibrosis in the obstructed segments of PUJ and older patients. The ICCs were reduced in the obstructed segment of PUJ as well as the DSM in a majority of the patients. Few patients had normal ICCs at the PUJ and surgical margins. There was a strong correlation between low renal function on nuclear scan, grade 4 fibrosis on Masson's trichrome stain, and absence of ICCs on c-Kit-stained slides.

Conclusions: On histological examination, the PUJ showed a narrow lumen, severe grades of fibrosis, muscle hypertrophy/atrophy, and low to absent ICCs, which correlate with severe obstruction and reduced renal function. Age is an important factor in the progression of the disease, and significantly higher grades of fibrosis were noted in older patients. Regular follow-up with USG and renal scan for recurrence of obstruction in the early postoperative period is of prime importance.

## Introduction

The mechanism of urine transport in the urinary tract was thought to be due to the auto-rhythmicity of smooth muscle cells [1]. However, over the past decade, research on neuronal activity in the urinary tract has revealed the role of interstitial cells of Cajal (ICCs) as pacemakers [2,3]. Murakumo et al. postulated a combined neurogenic and myogenic theory for pelvi-ureteric junction obstruction (PUJO) [4]. A decrease in smooth muscle cells at the PUJ, abnormal muscle orientation, and collagen deposition, with reduction in Cajal cells, were proposed to be the main causative factors for PUJO by Solari et al. and Yurtcu et al. [5,6]. Obstruction of the flow of urine leads to back-pressure and damage to the renal parenchyma. Pyeloplasty is the treatment of choice in cases of PUJO to prevent further renal damage. The diagnosis is made by pre-operative renal function assessment by nuclear scans and improvement in function is seen during post-operative follow-up [7,8]. The resected segment of PUJ on histology reveals varying degrees of fibrosis and the number of pacemaker cells that correlate with the degree of obstruction. There are studies where the non-obstructed segment had many ICCs, while the obstructed part showed sparse or none [7]. Inugala et al. studied the correlation between ICCs in the resected PUJs and the surgical outcome [9]. Koleda et al. studied the density of ICCs in operated PUJO patients, but their observations differed from the findings of other authors [10]. This background underscores the dilemma the clinicians may face during the diagnosis and management of PUJO patients. Despite advances in understanding the pathogenesis of PUJO, the diagnostic utility of ICC quantification and its relationship with renal function remains underexplored. To address this knowledge gap, our study aims to analyze the microanatomy of the PUJ and the distal surgical margin (DSM), quantify ICCs, and correlate these findings with preoperative renal function in patients with PUJO [11,12]. The objectives of this study are to compare the findings at the distal margin with those at the obstructed segment, and to correlate preoperative renal function with the severity of fibrosis and the absence or reduction of ICCs, thereby aiding clinicians in predicting surgical outcomes and the likelihood of recurrence during postoperative follow-up.

## Materials and methods

An inter-institutional cross-sectional analytical study was conducted in collaboration with the Paediatric Surgery and Anatomy departments. It was carried out after the approval of the Institutional Ethical Committee (IEC), BV(DU)MC&H/Sangli/IEC/381/20. The study was conducted for a period of two years from January 2020 to December 2021. Patients diagnosed with PUJO by clinical evaluation and imaging studies like USG and DTPA scan were included in the study. They underwent Anderson-Hynes dismembered pyeloplasty for intrinsic obstruction. The PUJO cases were included after receiving consent from the parents. The cases with extrinsic obstruction due to crossing vessels or associated congenital anomalies were excluded. The sample size was calculated based on the reported incidence of PUJO, which is approximately one in 1,000 to 2,000 neonates [9], and the desired sample size for a 99.99% CI was seven cases. However, this study included all 54 patients who underwent surgery for PUJO during the study period.

**Figure 1:**
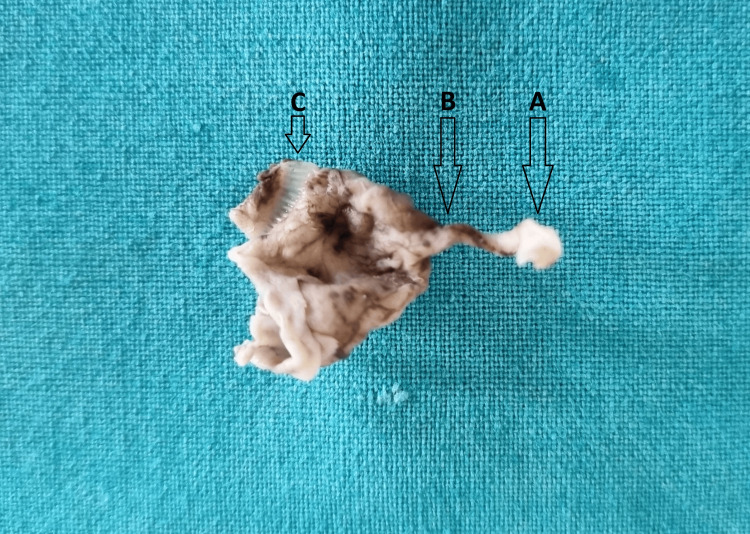
Resected segment of the PUJ showing normal DSM (A), a narrowed obstructed segment (B), and a dilated pelvis (C) PUJ, pelvi-ureteric junction; DSM, distal surgical margin

After surgery, the resected specimens of PUJs were placed in 10% formalin, correctly labeled, and transported to the Department of Anatomy. The gross features were noted to identify the dilated proximal pelvis, narrow obstructed middle part, and the normal distal end of the ureter (Figure 1).

Representative bits were taken. The tissue from the distal end was labeled as A-DSM, and the sample from the narrowed, obstructed middle segment was labeled as B-PUJ. Paraffin blocks were prepared using an automatic tissue processor. Thin sections measuring 5 to 7 µm were cut using a Leica microtome. The sections were stained with two different stains: routine hematoxylin and eosin (H&E; Merck) and Masson's trichrome stain (Yuccafine), following the manufacturers’ instructions.

Hematoxylin and eosin staining

The sections were deparaffinized and stained using the routine H&E staining procedure. The slides were observed under an Olympus Trinocular CX21i Biological microscope, and images were captured with an Olympus CCD camera (U-TV0.5XC-3). Processing of images was done using Magnus Pro 3.7 software. The histological findings were recorded, and measurements were taken using a stage micrometer and eyepiece reticle at 10× magnification. The corresponding lines of the micrometer scale and the micrometer eyepiece were observed and the value of the equidistant line was decided. On calculation, one division of the micrometer eyepiece was 4 μm [13]. The thickness of lamina propria (LP) from lining epithelium to muscle layer (ML) and ML thickness were recorded at three different places, and an average of the three readings was taken in micrometers [14].

Masson's trichrome staining

The sections were deparaffinized and hydrated using distilled water, mordanted in Bouin’s solution overnight, then stained with Weigert’s iron hematoxylin for 10 minutes, followed by Ponceau-Fuchsin for five minutes, and finally Aniline Blue for five minutes before mounting. The sections were examined under a scanner and low-power magnification to assess the proportion of smooth muscle and collagen fibers using differential staining. The dark blue-stained collagen was easily distinguished from the red-stained smooth muscle.

The degree of perifascicular collagen fibers and the proportion of smooth muscle were recorded in every section, and grading was assigned using a reference article [6,15]. The slides were labeled as Grade 1 fibrosis when no abnormality was noted; Grade 2 when there was mild perifascicular fibrosis with muscle hypertrophy; Grade 3 when moderate perifascicular fibrosis with muscle hypertrophy was seen; and Grade 4 when there was severe perifascicular fibrosis with muscle atrophy.

Immunohistochemistry

Immunohistochemistry (IHC) was performed using a c-kit antibody (Biogenex) for ICCs, as per the manufacturer’s instructions. Thin sections were deparaffinized and subjected to antigen retrieval. A peroxide block was applied for five minutes at room temperature, followed by a power block for five minutes, then the primary antibody and super enhancer. This was followed by poly-HRP (horseradish peroxidase), and the slides were counterstained with diaminobenzidine (DAB) chromogen and hematoxylin. The slides were mounted and viewed under an Olympus trinocular microscope at 40× magnification. Appropriate positive and negative controls were included. The ICCs were easily identifiable as spindle-shaped cells with brown cytoplasm seen in the subepithelial region and along the smooth muscle layers. The mast cells present in the wall naturally express positivity for c-kit staining; hence, they were used as a reliable internal control.

Ten consecutive high-power fields (hpf) were selected, and ICCs were counted and recorded. The average of three observations was taken for every slide, and grades were assigned as per reference articles [6,16]. Depending on the number of ICCs seen in 10 hpf, Grade 0 was assigned when the ICC count ranged from 0 to 1; Grade 1 for 2 to 5 ICCs; Grade 2 for 6 to 11 ICCs; and Grade 3 when more than 11 ICCs were seen in 10 hpf.

Statistical analysis

Statistical analysis was conducted using Microsoft Excel (Microsoft Corp., Redmond, WA, US) and SPSS-29 (IBM Corp. Released 2023. IBM SPSS Statistics for Windows, Version 29.0.2.0 Armonk, NY: IBM Corp).

Descriptive statistics

Mean and standard deviation were calculated for quantitative variables, such as the thickness of the LP and ML. Frequencies and percentages were obtained for qualitative attributes, including age, gender, blood urea nitrogen (BUN), serum creatinine, grading of fibrosis, and ICCs.

Inferential statistics

An unpaired t-test was applied (as the Shapiro-Wilk test was not significant, suggesting normal distribution) to compare different parameters of PUJ and DSM.

## Results

This study was conducted in 54 patients with PUJO. Of the total, 46 (85.2%) were male and eight (14.8%) were female. The patients’ ages ranged from 15 days to 13 years and were divided into three groups as shown in Table 1. The highest number of cases was in group 1, aged <12 months.

**Table 1:** Distribution of cases according to age and sex

Age Groups	Sex		Total
	Female	Male	
1 (<12 months)	6 (19.40%)	25 (80.60%)	31 (57.41%)
2 (13 to 60 months)	1 (7.70%)	12 (92.30%)	13 (24.07%)
3 (>61 months)	1 (10.00%)	9 (90.00%)	10 ((18.52%)
Total	8 (14.80%)	46 (85.20%)	54 (100.00%)

In 36 (66.7%) cases, the PUJO was detected antenatally. Among the patients who were not diagnosed antenatally, nine presented with pain, eight had urinary tract infection, and one had a lump in the abdomen. Out of the 54 cases, 37 (68.51%) had left-sided PUJO.

The BUN level ranged from 16 mg/dL to a maximum of 48 mg/dL, with an average of 23.55±7.22 mg/dL in group 1, 23.15±5.61 mg/dL in group 2, and 23.70±4.74 mg/dL in group 3. Using the standard reference levels of BUN as a benchmark, elevated urea levels were observed in 34 (62.96%) patients [17].

The serum creatinine values ranged from 0.3 to 1.1 mg/dL, with a mean of 0.61±0.15, 0.61±0.10, and 0.67±0.13 in the three age groups, respectively. Based on standard creatinine reference levels [17], 33 (61.11%) patients had increased creatinine levels. Distribution of patients with normal and elevated BUN and serum creatinine is shown in Table 2.

**Table 2:** BUN and serum creatinine in the study groups Reference range for BUN in all age groups is 10-20 mg/dL; reference range for creatinine is 0.2-0.4 mg/dL for Group 1, 0.3-0.7 mg/dL for Group 2, and 0.5-1 mg/dL for Group 3 BUN, blood urea nitrogen

Age Groups	BUN		Serum Creatinine	
	No. of Patients With Normal Values	No. of Patients With Abnormal Values	No. of Patients With Normal Values	No. of Patients With Abnormal Values
1 (<12 months)	14 (45.16 %)	17 (54.84%)	1 (3.23%)	30 (96.77%)
2 (13 to 60 months)	4 (30.77%)	9 (69.23%)	13 (100%)	0
3 (>61 months)	2 (20%)	8 (80%)	7 (70%)	3 (30%)
Total	20 (37.04%)	34 (62.96%)	21 (38.89%)	33 (61.11%)

Hematoxylin & eosin stain

**Figure 2:**
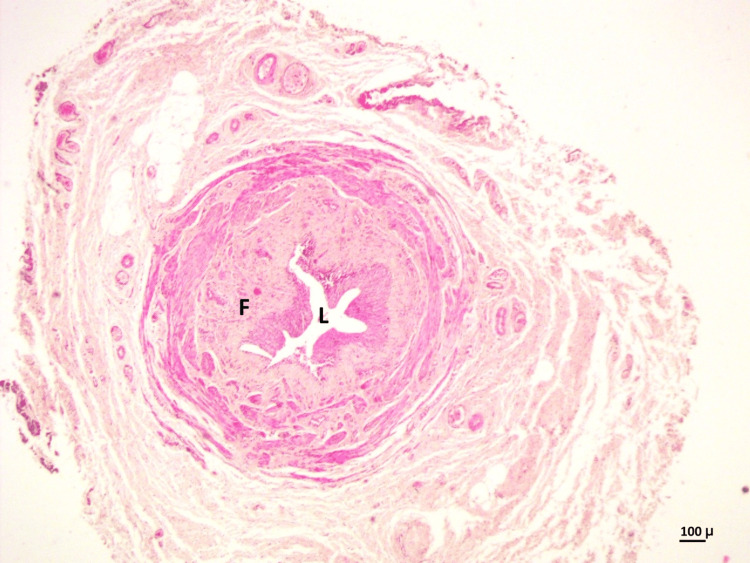
PUJ showing a narrow lumen (L), lined by transitional epithelium, and increased fibrosis (F) in the submucosa and wall (H&E stain, 10×) PUJ, pelvi-ureteric junction; H&E, hematoxylin and eosin

The histological parameters were studied in H&E-stained slides at the PUJ and DSM. The PUJ exhibited a narrow lumen, lined by transitional epithelium that was thickened in some cases and normal in others. There was increased fibrosis in the submucosa and ureteric wall (Figure 2).

**Figure 3:**
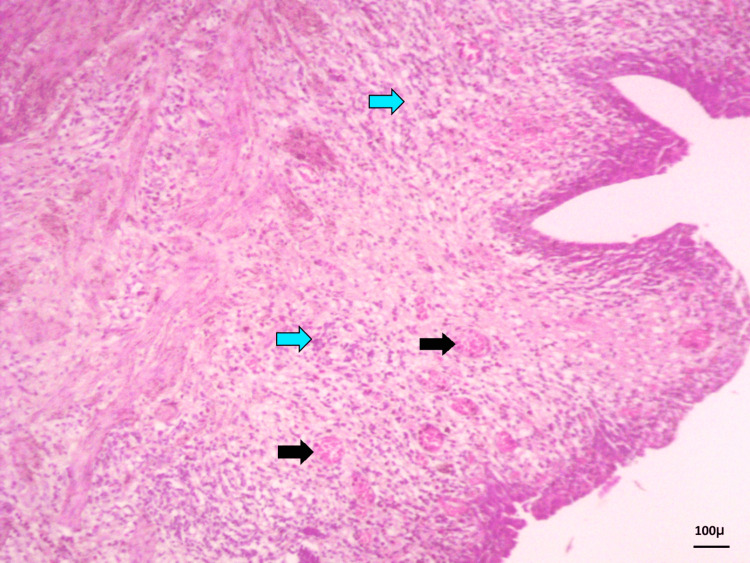
Ureteric wall showing increased vascularity (black arrows) and lymphocytic infiltration (blue arrows) (H&E stain, 10×) H&E, hematoxylin and eosin

In 16 cases, the ureteric wall showed increased vascularity and lymphocytic infiltration, suggestive of inflammation. Of these 16, 12 belonged to group 1, and two each to groups 2 and 3 (Figure 3).

Prominent nerve bundles were noted in six cases, of which five were from group 1 and one case from group 3. Severe submucosal fibrosis was seen in one case, aged eight years, which belonged to group 3.

**Figure 4:**
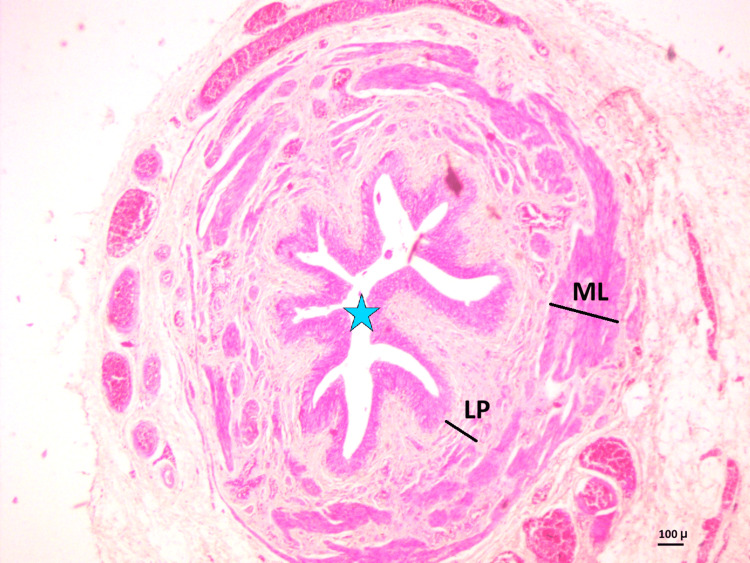
DSM with star-shaped normal lumen (blue star) and preserved lining epithelium (H&E stain, 10×) DSM, distal surgical margin; LP, thickness of lamina propria; ML, thickness of muscle layer

In all sections, the DSM showed a normal star-shaped ureteric lumen lined by transitional epithelium. The wall showed hypertrophied smooth muscle and varying degrees of fibrosis. The thickness of the LP and ML was measured as shown in Figure 4.

The average thickness of the LP at the PUJ was 170.77 µm, and at the DSM was 154.97 µm. The ML thickness was 637.37 µm at the PUJ and 535.11 µm at the DSM.

The thickness of the LP at the PUJ and DSM across the three age groups is shown in Table 3. There is a statistically significant difference in LP thickness at the PUJ and DSM in group 3, while the difference is not significant in groups 1 and 2 (Table 3).

**Table 3:** Comparison of the thickness of LP at PUJ and DSM on H&E stain PUJ, pelvi-ureteric junction; DSM, distal surgical margin; LP, lamina propria; H&E, hematoxylin and eosin

Age Groups	Average LP Thickness at PUJ (um)	Average LP Thickness at DSM (um)	P-value
1 (<12 months)	162.11±48.93	148.68±46.93	0.27
2 (13 to 60 months)	174.12±54.55	162.01±41.39	0.33
3 (>61 months)	193.29±54.87	165.33±43.19	0.029

The thickness of the ML at the PUJ and DSM in the three groups is shown in Table 4. There is a statistically significant difference in ML thickness at the PUJ and DSM in group 3, while the difference is not significant in groups 1 and 2 (Table 4).

**Table 4:** Comparison of the thickness of ML at PUJ and DSM on H&E stain PUJ, pelvi-ureteric junction; DSM, distal surgical margin; LP, lamina propria; H&E, hematoxylin and eosin; ML, muscle layer

Age Groups	Average Thickness of ML at PUJ (um)	Average Thickness of ML at DSM (um)	P-value
1 ( 12 months)	595.55±264.22	495.66±241.82	0.13
2 (13 to 60 months)	634.71±167.69	598.88±201.75	0.45
3 (>61 months)	770.47±192.12	574.5±234.23	0.000

Masson's trichrome stain

**Figure 5:**
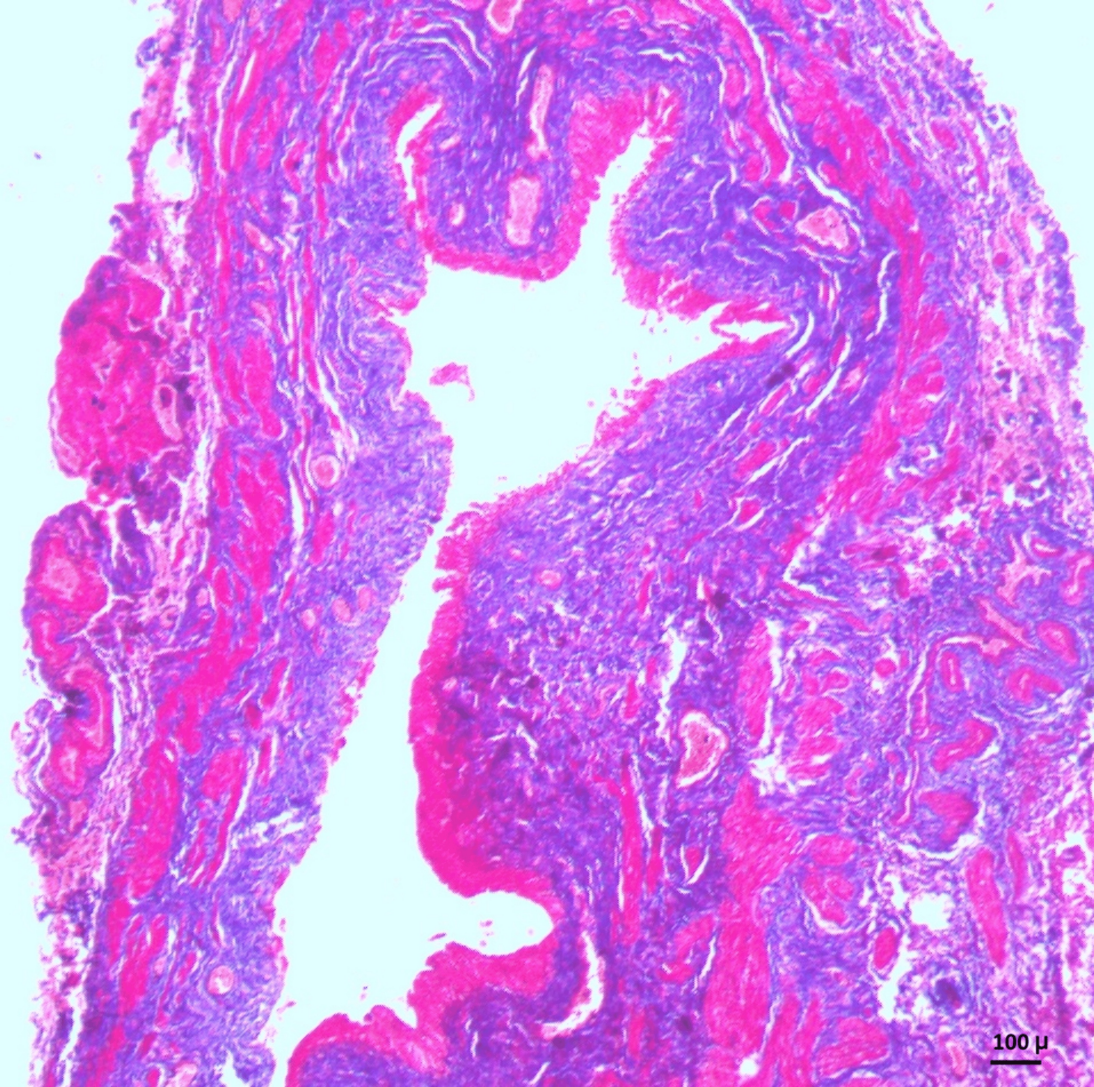
Grade 4 fibrosis with thick perifascicular collagen bundles and atrophy of muscle fibers (Masson's trichrome, 10×; blue - collagen bundles, red - smooth muscle)

The degree of perifascicular fibrosis and muscle hypertrophy or atrophy was assessed in Masson's trichrome-stained slides at the DSM and PUJ, based on which grading was done. The perifascicular fibrosis in the ureteric wall appeared as thick collagen bundles stained blue, while muscle fibers were identified as pink-to-red-stained fascicles. Figure 5 shows grade 4 fibrosis with severe perifascicular collagen and atrophy of smooth muscle. Fibrosis was more marked at the obstructed PUJ than at the DSM in all three age groups. In one case, definitive submucosal fibrosis was noted.

As shown in Table 5, severe fibrosis of grade 3 (n=19) and grade 4 (n=33) was observed in 52 cases (96.30%) at the PUJ.

**Table 5:** Distribution of cases based on Masson's trichrome grading at PUJ PUJ, pelvi-ureteric Junction

Grades of Fibrosis on Masson's trichrome at PUJ	Age Groups			
	1 (<12 months)	2 (13 to 60 months)	3 (>61 months)	Total
Grade 1	0	0	0	0
Grade 2	2 (6.45%)	0	0	2 (3.70%)
Grade 3	10 (32.26%)	4 (30.77%)	5 (50%)	19 (35.19%)
Grade 4	19 (61.29%)	9 (69.23%)	5 (50%)	33 (61.11%)
Total	31 (100%)	13 (100%)	10 (100%)	54 (100%)

At the DSM (Table 6), grade 3 (n=17) and grade 4 (n=21) fibrosis were noted in 38 cases (70.37%). Tables 5 and 6 also show that higher grades of fibrosis were seen as age advances.

**Table 6:** Distribution of cases based on Masson's trichrome grading at DSM DSM, distal surgical margin

Grades of fibrosis on Masson's trichrome at DSM	Age Groups			
	1 (<12 months)	2 (13 to 60 months)	3 (>61 months)	Total
Grade 1	1 (3.23%)	1 (7.69%)	0	2 (3.70%)
Grade 2	9 (29.03%)	3 (23.08%)	2 (20%)	14 (25.93%)
Grade 3	7 (22.58%)	6 (46.15%)	4 (40%)	17 (31.48%)
Grade 4	14 (45.16%)	3 (23.08%)	4 (40%)	21 (38.89%)
Total	31 (100%)	13 (100%)	10 (100%)	54 (100%)

Immunohistochemistry using C-kit antigen

**Figure 6:**
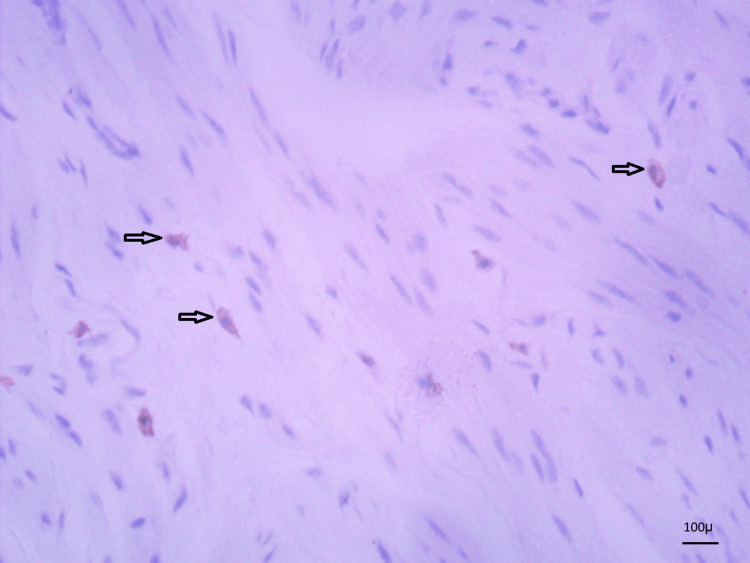
C-kit-stained slide showing ICCs (spindle cells with granular brown cytoplasm, indicated by black arrows) (IHC, 40×) ICCs, interstitial cells of Cajal; IHC, immunohistochemistry

The ICCs were noted on IHC-stained slides. The cells appeared spindle-shaped with brown cytoplasm along the subepithelial and smooth MLs, as shown in Figure 6.

The interstitial cells were counted, and grading was done. As shown in Table 7, at the PUJ, 43 (79.63%) of the cases had grade 0 ICCs. 

**Table 7:** ICCs grading at PUJ on c-kit stained slides (IHC) ICCs, interstitial cells of Cajal; IHC, immunohistochemistry; PUJ, pelvi-ureteric Junction

Grades of ICC on C-kit Stain at PUJ	Age Groups			Total
	1 (<12 months)	2 (13 to 60 months)	3 (>61 months)	
Grade 0	26	9	8	43 (79.63%)
Grade 1	3	1	1	5 (9.26%)
Grade 2	1	2	1	4 (7.41%)
Grade 3	1	1	0	2 (3.70%)
Total	31	13	10	54 (100%)

It is evident from Table 8 that at the DSM, 39 (72.22%) patients had grade 0 ICCs.

**Table 8:** ICCs grading at DSM on c-kit stained slides (IHC) ICCs, interstitial cells of Cajal; IHC, immunohistochemistry; PUJ, pelvi-ureteric Junction

Grades of ICC on C-kit Stain at PUJ	Age Groups			Total
	1 (<12 months)	2 (13 to 60 months)	3 (>61 months)	
Grade 0	25	7	7	39 (72.22%)
Grade 1	4	2	2	8 (14.81%)
Grade 2	1	2	0	3 (5.56%)
Grade 3	1	2	1	4 (7.41%)
Total	31	13	10	54 (100%)

Correlation of renal function with histological and immunohistochemical parameters

Preoperative DTPA renal scans showed impaired renal function and an obstructive curve on the affected side in all cases. To assess the correlation between preoperative renal function and histological parameters, the cases were divided into three groups based on the percentage of renal function on the preoperative DTPA scan: group A: renal function 0-30%; group B: renal function 31-40%; group C: renal function >40%.

Severe fibrosis (grade 4 on Masson's trichrome stain) and absence of ICCs (grade 0 on C-kit stain) at the PUJ were compared across the three groups. Among patients with low renal function on preoperative scans, 75% had grade 4 fibrosis as well as grade 0 ICC. In the group with >40% renal function, 54.54% had severe fibrosis and 72.72% had no ICCs (Table 9).

**Table 9:** Correlation of preoperative renal function by DTPA scan with grade 4 fibrosis (Masson's trichrome stain) and grade 0 ICC (C-kit stain) Group A, renal function 0-0%; Group B, renal function 31-40%; Group C, renal function >40%; ICC, interstitial cell of Cajal

Groups based on renal function	Fibrosis grade 4	ICC grade 0
Group A (n=8)	6 (75%)	6 (75%)
Group B (n=13)	9 (69.23%)	8 (61.54%)
Group C (n=33)	18 (54.54%)	24 (72.72%)

## Discussion

This study included 54 patients diagnosed with PUJO who underwent surgery. A male preponderance was observed, with 85.2% of the patients being male, a trend similarly noted in other studies by various authors [8,15,16]. The biochemical parameters, such as BUN and serum creatinine, were within normal limits or slightly elevated, as unilateral PUJO typically does not significantly impair these values.

In our study, PUJO was antenatally detected in 36 cases (66.7%), whereas Moriera Pinto et al. [8] reported a detection rate of 74%. The lower detection rate in our region may be attributed to ignorance, limited accessibility, and the unaffordability of advanced imaging in rural areas. Among patients not diagnosed antenatally, abdominal pain was the most common presenting symptom, followed by urinary tract infection (UTI). Moriera Pinto et al. [8] also reported similar presenting complaints in their study.

Histological examination using H&E staining revealed differences between the PUJ and DSM. At the PUJ, the ureteric lumen appeared narrow, and the lining epithelium was either thickened or normal. The wall showed hypertrophied smooth muscles and increased fibrosis, more prominent at the PUJ than the DSM. There was also increased vascularity and lymphocytic infiltration, suggesting inflammation, which may progress to fibrosis and worsen the obstruction. Prominent nerve bundles were seen in some cases, indicating increased peristaltic effort by the ureters to overcome the obstruction. Nandan et al. compared PUJO cases with age-matched controls (patients operated on for Wilms tumor) and found greater muscle hypertrophy and fibrosis in the obstructed segments than in the resected margins, findings similar to ours [16]. In a few cases, the wall showed atrophied smooth muscles and severe perifascicular fibrosis, which was again more prominent at the PUJ. Murakamo et al. described similar features in the obstructed segment, including preserved lining epithelium, inflammation, thin and sparse muscle fascicles, and accumulated connective tissue in the interfascicular spaces [4]. Miranda et al. [12], in a literature review, also noted similar findings.

In a study by Zhang et al. [15], the average PUJ muscle thickness in controls of the same age group was 0.3±0.05 mm. In our study, the average was 0.535 mm, indicating significant hypertrophy. Notably, there was a statistically significant difference in the thickness of the LP and ML at the PUJ and DSM in group 3 (patients aged over five years). There was no significant difference in ML thickness at younger ages, but after five years, the PUJ remained thick while the distal segment underwent atrophy. This suggests that older patients may have compromised postoperative function of the distal segment, potentially affecting surgical outcomes.

Masson's trichrome staining proved effective in detecting and grading fibrosis due to its differential staining characteristics. The present study demonstrated higher grades of fibrosis in obstructed PUJ segments, especially among older patients. Similar findings were reported in studies by Muslim Yurtçu et al., Cancian M et al., and Hang PL et al. [6,14,15].

ICCs were markedly reduced at both the PUJ and DSM in all patients. This aligns with previous studies that reported low to absent ICCs as a contributing factor in obstruction [5-7,9,12,18]. Some cases showed hypertrophied nerve bundles and an adequate number of ICCs. Koleda et al. reported normal to increased ICC numbers at obstruction sites, which contrasts with many other studies. They suggested this may represent a compensatory pacemaker response to obstruction. They also noted that like intestinal ganglion cells, ureteric pacemaker cells respond to inflammation and their numbers decline with age [10].

A key observation in our study was a strong correlation between poor preoperative renal function (as shown on DTPA scan), grade 4 fibrosis (Masson's trichrome), and absence of ICCs (C-kit stain). Interestingly, even some patients with over 40% renal function preoperatively exhibited severe fibrosis and reduced ICCs, emphasizing the importance of clinical judgment and early intervention to preserve renal function in PUJO cases. Tharanendran et al. similarly concluded that pyeloplasty performed between one and three months of age offers the best chance for renal recovery [19]. Although severe fibrosis and ICC depletion were more common at the PUJ, some DSMs also showed similar features. This could contribute to late-onset distal fibrosis, reinforcing the need for long-term follow-up using ultrasound and renal scans in the early postoperative period.

The correlation between preoperative renal scans and histological changes is a major strength of this study, offering a basis for guiding postoperative monitoring. While postoperative renal scans and functional comparisons would have provided further insight, these data could not be included and remain a limitation of this study, as well as a potential area for future research.

## Conclusions

The microanatomy of the resected PUJ revealed differences between the site of obstruction and the distal margin of the ureter. The PUJ exhibited histological features such as a narrow lumen, severe grades of fibrosis, muscle hypertrophy or atrophy, and low to absent ICCs, all of which correlate with severe obstruction and reduced renal function.

Age is an important factor in disease progression, with significantly higher grades of fibrosis observed in older patients. The findings in this study underscore the importance of antenatal screening, early postnatal evaluation, and timely intervention to achieve favorable outcomes in patients with PUJO. Regular follow-up of operated patients with USG and renal scans is crucial for detecting obstruction or recurrence in the early postoperative period.
